# Dual-Encoder UNet-Based Narrowband Uncooled Infrared Imaging Denoising Network

**DOI:** 10.3390/s25051476

**Published:** 2025-02-27

**Authors:** Minghe Wang, Pan Yuan, Su Qiu, Weiqi Jin, Li Li, Xia Wang

**Affiliations:** MOE Key Laboratory of Optoelectronic Imaging Technology and System, Beijing Institute of Technology, Beijing 100081, China; 3220215088@bit.edu.cn (M.W.); 3120185348@bit.edu.cn (P.Y.); jinwq@bit.edu.cn (W.J.); lili@bit.edu.cn (L.L.); angelniuniu@bit.edu.cn (X.W.)

**Keywords:** uncooled infrared, infrared focal plane detector, thermal imaging denoising, dual-encoder UNet

## Abstract

Uncooled infrared imaging systems have significant potential in industrial hazardous gas leak detection. However, the use of narrowband filters to match gas spectral absorption peaks leads to a low level of incident energy captured by uncooled infrared cameras. This results in a mixture of fixed pattern noise and Gaussian noise, while existing denoising methods for uncooled infrared images struggle to effectively address this mixed noise, severely hindering the extraction and identification of actual gas leak plumes. This paper presents a UNet-structured dual-encoder denoising network specifically designed for narrowband uncooled infrared images. Based on the distinct characteristics of Gaussian random noise and row–column stripe noise, we developed a basic scale residual attention (BSRA) encoder and an enlarged scale residual attention (ESRA) encoder. These two encoder branches perform noise perception and encoding across different receptive fields, allowing for the fusion of noise features from both scales. The combined features are then input into the decoder for reconstruction, resulting in high-quality infrared images. Experimental results demonstrate that our method effectively denoises composite noise, achieving the best results according to both objective metrics and subjective evaluations. This research method significantly enhances the signal-to-noise ratio of narrowband uncooled infrared images, demonstrating substantial application potential in fields such as industrial hazardous gas detection, remote sensing imaging, and medical imaging.

## 1. Introduction

Infrared imaging technology is widely used in military detection, industrial exploration, civilian temperature measurement, and other applications. In particular, it has seen further development in recent years in fields such as industrial gas leakage detection [1]. However, correction of the inherent pattern noise in infrared focal plane imaging has always been a critical issue [2], especially because the sensitivity of uncooled infrared detectors is relatively low. Additionally, factors such as the use of narrow bandpass filters in gas leakage detection increase the severity of the temporal and spatial stripe noise of thermal images, thereby limiting their application in various scenarios. Therefore, infrared image denoising technology to reduce noise in uncooled infrared images and improve the signal-to-noise ratio (SNR) has become an important research direction.

**Figure 1:**
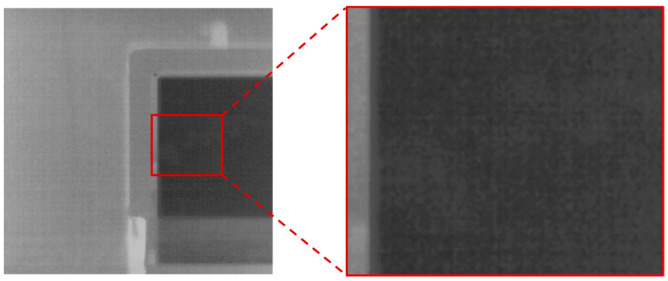
Noise characterization of narrowband infrared images.

Infrared image denoising is a fundamental task in infrared image processing technology, with the goal of eliminating noise components from infrared images. At present, the technology for uncooled infrared imaging systems has reached maturity. To correct the non-uniformity of the detector, most non-uniformity noise can typically be effectively removed by dynamically correcting bias drift using internal baffles, based on prior laboratory blackbody calibrations. However, this correction approach does not address the calibration parameter shifts introduced by narrowband filters after the system is integrated. In the residual noise components of the initially corrected narrowband uncooled infrared images, the noise types that primarily reduce the image signal-to-noise ratio include Gaussian random noise and spatiotemporal line stripe noise [3,4,5,6], which can interfere with the detection of weak gas targets (as shown in Figure 1). Given the relatively high proportion of stripe noise in early uncooled infrared cameras, infrared image denoising technology has largely concentrated on removing stripe noise. This includes methods based on spatial filters [7], transform domain filtering methods [8], and targeted modeling techniques based on noise characteristics [9,10,11], all of which demonstrate varying degrees of effectiveness in stripe noise removal.

Currently, there is limited research on methods that simultaneously remove temporal and spatial noise from infrared images, primarily due to the difficulty of modeling both types of noise concurrently. However, Hu et al. have made progress in denoising research using deep learning methods [12], demonstrating that mature visible light image denoising neural networks are also effective in learning and eliminating complex noise in infrared images. In the realm of deep learning-based image denoising technology, the DnCNN network introduced by K. Zhang et al. [13] has surpassed traditional denoising methods like BM3D [14] and WNNM [15] in terms of performance. The UNet architecture, which is based on a residual CNN, is capable of learning noise characteristics and removing complex noise [16]. However, due to the limited receptive field of CNN networks, their ability to remove noise is constrained. With the rise of Transformer technology in computer vision, self-attention mechanisms have been integrated into image denoising, resulting in numerous image restoration networks that enhance denoising performance benchmarks [17,18,19]. However, because image denoising methods in the visible light domain have not been specifically designed to tackle the issue of mixed Gaussian noise and stripe noise, their denoising capabilities fail to meet the necessary requirements.

To address these challenges, we propose DER-UNet, an infrared image denoising network featuring a dual-encoder UNet structure incorporating two types of scale attention mechanisms. The proposed network, based on the basic residual UNet denoising residual network, incorporates an encoder branch with extended-scale attention to better learn the row–column features of stripe noise. The main structure of the DER-UNet network comprises a UNet based on a basic scale residual attention (BSRA) encoder–decoder, an enlarged scale residual attention (ESRA) encoder branch, and an encoding feature fusion module. The features extracted from the two encoder branches are combined and connected to the decoder layer through the residual dense network structure to refine the effective features. The experimental results demonstrate that DER-UNet achieves the best uncooled infrared image denoising results reported, and ablation experiments prove the effectiveness of the network structure.

The contributions of the proposed method are summarized as follows:

(1) To address the differences in characteristic scales between Gaussian noise and stripe noise, we designed a dual-branch encoder UNet structure based on the traditional residual UNet network, which considers the features of both noise types. Additionally, we employed a feature fusion structure based on the RCAG module to effectively integrate the two noise characteristics. The resulting network structure is concise and demonstrates outstanding performance, achieving the best denoising results among existing methods.
(2) We proposed an expanded scale encoder branch design specifically for learning the characteristics of stripe noise. To reduce the computational burden associated with this expanded scale encoder branch, we utilized depthwise separable convolutions in place of traditional convolution operations, significantly lowering the computational load while maintaining effective learning capabilities.

## 2. Related Work

### 2.1. Infrared Image Denoising Technology

Infrared image denoising technology aims to remove various types of noise from infrared images and enhance their SNRs. In traditional mixed noise denoising methods, the main approach involves modeling based on the image characteristics of stripe noise and then using image processing techniques that can extract stripe noise for denoising.

Song et al. proposes a method based on the expectation maximization (EM) framework that divides the overall stripe and noise removal problem into two independent sub-problems [20]. Specifically, the conditional expectation of the true image is calculated based on observations from the previous iteration and estimated stripes, and the column mean of the residual image is estimated to ensure maximum likelihood estimation (MLE). He et al. introduced spatial priors of stripes into the mixed denoising model and, for the first time, introduced frequency-domain priors in plug-and-play technology, which is beneficial for the mutual enhancement of multi-domain feature constraints in stripe removal [21]. In another study, wavelet functions were used to extract the approximate and vertical components of the original image containing stripe noise [22], denoise the approximate components through parameter estimation, and denoise the vertical components through guided filtering; wavelet reconstruction was performed to achieve denoising of the original image.

With the development of deep learning technology, the denoising of complex infrared images based on neural networks has made significant progress. Several neural networks adapted for infrared image denoising have been developed by designing network structures with powerful feature-learning and image restoration capabilities. A CNN architecture was adopted along with second-order attention mechanisms and non-local modules at the regional level to improve the extraction of image features and fit noise residuals [3]. Yang et al. proposes an infrared image denoising method based on multi-level feature attention networks (MLFANs) with adversarial learning [4]. The multi-level feature attention block (MLFAB) aims to extract features from different levels and establish connections through feature fusion to enhance the details of the reconstructed image, and a discriminator is developed to provide adversarial learning loss. Hu et al. proposes an infrared thermal image denoising method based on residual learning and a symmetric multi-scale (SM) decoder sampling structure (SMEDS) [12]. The U-shaped SMEDS aims to extract SM information from different layers and focuses on decoding recovery. Currently, infrared image denoising is primarily based on CNNs, which lack denoising network designs based on Transformer structures.

### 2.2. Dual-Encoder UNet

UNet first appeared in the field of medical image segmentation [23], and has become a fundamental structure in the field of image denoising, demonstrating strong encoding and decoding capabilities. The design of dual-encoder branches has been studied in various domains, such as semantic segmentation, saliency detection, image denoising, image enhancement, and image super-resolution [24]. The dual-encoder UNet was developed from the conventional UNet by adding an additional encoder branch to the left side of the UNet, providing multiple attentions for better exploration of the target features.

Fu et al. designed a dual-encoder feature attention medical image segmentation network, DEAU-UNet [25], which uses dual encoders in the encoding stage to independently achieve macro- and micro-feature extraction, followed by feature attention fusion. This enables the network to perform well in the recognition of macro-features and shows significant improvement in the processing of micro-features. Liang et al. designed a medical image segmentation network, N-Net, proposing a dual-encoder model based on the UNet network to deepen the network depth and enhance feature extraction capabilities [26]. Channel-level global features were obtained by adding Squeeze-and-Excitation (SE) modules to the dual-encoder model, and the introduction of full-size skip connections promoted the fusion of low-level details and high-level semantic information. Li et al. proposed an Efficient Residual Double-Coding UNet (ERDUnet), which also constructs a dual-encoder UNet for medical image segmentation [27]. It improved feature extraction efficiency by simultaneously extracting local features and global continuity information through the encoder module. Yao et al. proposed a dual-encoder UNet called DEUNet for high dynamic range (HDR) image denoising and reconstruction [28]. It learned brightness and texture information through two feature extraction branches for HDR image reconstruction. The two branches interact through spatial feature transformation, fully utilizing multi-scale information at different image levels. Moreover, the network includes another decoding network for fusing the image brightness and texture information, as well as a weighted network to selectively retain the most useful information.

### 2.3. Multi-Scale Attention

Neural networks based on CNNs have a receptive field that is related to the size of the convolutional kernels and depth of the network. Since denoising networks typically use small 3 × 3 convolutional kernels to balance feature extraction effectiveness and computational load and are usually limited in depth, they can only perform local perception on input images. Moreover, as the receptive field expands, the perception ability decreases. Multi-scale attention mechanisms have been developed to enhance the receptive field of CNNs, and research progress has been made in fields such as target detection [29], image dehazing [30], and image super-resolution [31].

In the field of image denoising, a Multi-Scale Residual Dense Cascade UNet (MCU-Net) that uses Multi-Scale Residual Dense Blocks (MRDBs) to connect the encoder and decoder of the UNet has been proposed [32]. Compared with skip connections, block connections using MRDB can adaptively transform the features of the encoder and transfer them to the UNet decoder. Gou et al. proposed a novel multi-scale adaptive network (MSANet) for single-image denoising that simultaneously contains intra-scale features and cross-scale complementarity [33]. An adaptive multi-scale block (AMB) can expand the receptive field and aggregate multi-scale information, whereas an Adaptive Fusion Block (AFuB) is dedicated to fusing multi-scale features from coarse to fine. Thakur et al. proposed a blind Gaussian denoising network for designing a dual-path model [34]. One path uses a Multi-Scale Pixel Attention (MSPA) block, and the other path uses a Multi-Scale Feature Extraction (MSFE) block.

## 3. Dual-Domain Perception Denoising Network

### 3.1. Narrowband Uncooled Infrared Noise Model

The noise components in uncooled infrared images primarily consist of temporal Gaussian noise and spatiotemporal striping noise. Upon the introduction of a narrowband filter, the image signal is diminished, leading to a further reduction in the image signal-to-noise ratio. Considering these two types of noise as additive noise, the degraded image y can be expressed as

(1) $$y=u+n_{g}+s$$

where u is a noise-free clean image, $n_{g}$ denotes additive white Gaussian noise, and s denotes the stripe noise.

Relative to the removal of Gaussian noise, the characteristics of composite noise in uncooled infrared images are more complex, and the scale ranges of Gaussian and stripe noises are different. To correct complex infrared noise while effectively preserving weak gas plume signals, it is necessary to utilize the learning and fitting capabilities of neural networks to remove composite noise.

### 3.2. Dual-Branch Encoder UNet Network Structure

**Figure 2:**
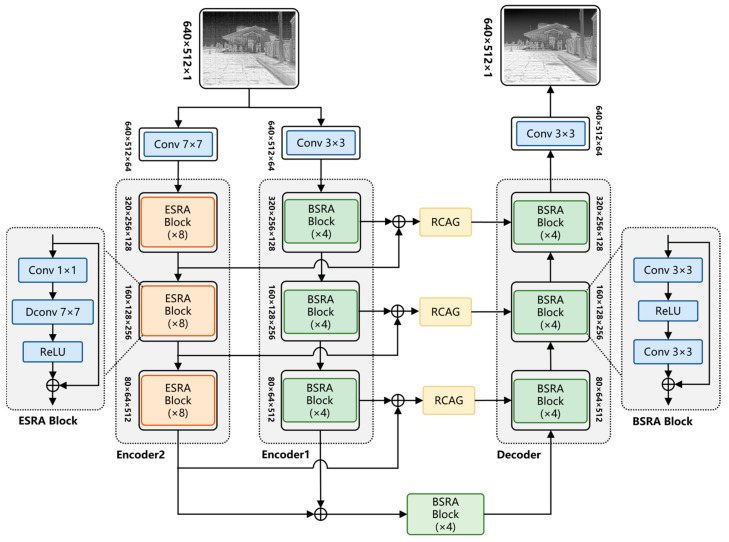
Architecture of DER-UNet.

The proposed DER-UNet network is designed based on a dual-encoder UNet architecture that consists of two encoder branches with different scales and one decoder branch, as shown in Figure 2. To address the problem of different feature scales for Gaussian noise and stripe noise, two types of convolutional encoder branches with different scales are designed: the BSRA encoder for learning the features of Gaussian noise and the ESRA encoder for learning the features of stripe noise. The features obtained from the two encoder branches are combined and connected to the decoder layer through the feature fusion connection structure. The feature fusion connection structure can further fuse the features obtained by the encoders and, to some extent, predict and restore the image, thereby enhancing the denoising effect.

**Figure 3:**
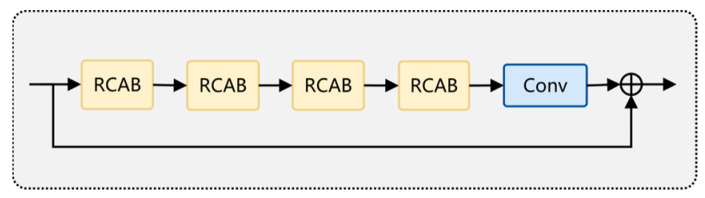
Structure of RCAG module.

The detailed structure of the DER-UNet network is as follows:

(1) Three-layer Residual UNet Structure: The basic structure of the network is a three-layer downsampling residual UNet network with a BSRA encoder and decoder designed as symmetrical structures. The BSRA encoder is based on a 3 × 3 residual convolution kernel, as shown in Figure 2, with each layer of the encoder structure stacking four layers of BSRA blocks. The tensors obtained at each layer of the two encoder branches are of the same scale, with downsampling and upsampling designs between the layers. The numbers of feature channels from top to bottom are 64, 128, and 256, respectively. Upon obtaining the features from the two encoders at each layer, they are added together and sent to the corresponding layer of the decoder.
(2) ESRA Encoder: To effectively learn the large-scale features of stripe noise, an Enlarge Scale Attention encoder branch is designed using a 7 × 7 convolution kernel to construct the ESRA blocks. Depthwise separable convolution is used instead of conventional convolution to reduce the computational load of the large-sized convolutional kernel, as shown in Figure 2. The convolution structure of each basic module contains depthwise separable convolution layers and ReLU layers, with the input connected to the output through a residual connection.
(3) Feature Fusion Connection Module: Further fusion processing is required because a difference exists in the scale of the features obtained from the two encoder branches. Therefore, on the basis of the skip connection in the conventional UNet network, the residual channel attention group (RCAG) module proposed by the residual channel attention network (RCAN) [35] is added. The RCAG structure is developed by cascading four residual channel attention blocks (RCABs) and further fusing and reconstructing the mixed features from the two encoder branches. The structure of the RCAG module is shown in Figure 3.

The network’s processing pipeline can be mathematically characterized as follows: Initially, an image I corrupted by noise is provided as input and a convolution operation is applied to augment the dimensionality of the feature space. The computational process is expressed as

(2) $$\begin{array}{l} X_{0}^{\mathrm{BSRA}}={\mathrm{Conv}}_{3\times 3}(I)\\ X_{0}^{\mathrm{ESRA}}={\mathrm{Conv}}_{7\times 7}(I) \end{array}$$

Subsequently, the BSRA encoder employs a three-level coding strategy that incorporates downsampling. Since each encoder layer is constructed from a series of cascaded encoder modules, the representations for the basic BSRA block and the ESRA block are given by

(3) $$\begin{array}{l} \mathrm{BSRA}(x)={\mathrm{Conv}}_{3\times 3}(\sigma_{R}({\mathrm{Conv}}_{3\times 3}(x)))+x\\ \mathrm{ESRA}(x)=\sigma_{R}({\mathrm{DConv}}_{7\times 7}({\mathrm{Conv}}_{1\times 1}(x)))+x \end{array}$$

where $\sigma_{R}$ denotes the activation function ReLU, ${\mathrm{DConv}}_{7\times 7}$ represents a depthwise separable convolution with a 7 × 7 kernel.

The input feature map at each level of the multi-layer encoder is obtained from the output of the previous level. Define $X_{i}^{\mathrm{BSRA}}\in {\mathbb{R}}^{C\times H\times W}$ as the input feature map to each encoder level, where C, H, and W represent the number of channels, height, and width, respectively. Consequently, the output of each layer of the BSRA encoder can be expressed as

(4) $$X_{i+1}^{\mathrm{BSRA}}=\mathrm{Downsample}({\mathrm{BSRA}}^{m}(X_{i}^{\mathrm{BSRA}}))$$

where i∈1,2,3, ${\mathrm{BSRA}}^{m}$ represents a composite module executed in series m, and Downsample represents a downsampling operation with a stride of 2.

Following a similar approach, the ESRA encoder utilizes extended three-level downsampling. If $X_{i}^{\mathrm{ESRA}}\in {\mathbb{R}}^{C\times H\times W}$ represents the input feature map for each level, then the respective output for each level is

(5) $$X_{i+1}^{\mathrm{ESRA}}=\mathrm{Downsample}({\mathrm{ESRA}}^{n}(X_{i}^{\mathrm{ESRA}}))$$

Following the final downsampling stage, the BSRA module integrates the resulting features.

(6) $$X_{fusion}={\mathrm{BSRA}}^{m}(X_{i}^{\mathrm{BSRA}}+X_{i}^{\mathrm{ESRA}})$$

The network then proceeds with iterative upsampling. Each upsampling layer integrates the outputs of the dual encoders with features received from the higher-resolution decoder layer. The mathematical representation of this upsampling module can be expressed as

(7) $$U_{i-1}=\mathrm{Upsample}({\mathrm{BSRA}}^{m}(U_{i}+\mathrm{RCAG}(X_{i}^{\mathrm{BSRA}}+X_{i}^{\mathrm{ESRA}}))$$

where Upsample represents an upsampling operation using transposed convolution.

A convolutional operation is then applied to the upsampled feature maps for channel restoration, generating the final predicted image.

(8) $$Y={\mathrm{Conv}}_{3\times 3}(U)$$

### 3.3. Loss Function and Training Process

Network loss is generated from the ground-truth and estimated images. The loss function used is based on the L1 loss and incorporates the total variation (TV) loss as a regularization term, specifically.

(9) $$Loss(u,v)=L_{MVE}(u,v)+\lambda L_{tv}(u,v)$$

where $L_{MVE}$ represents the L1 loss; λ represents the regularization coefficient for the TV loss $L_{\mathrm{tv}}$, used to adjust the regularization strength; u represents the ground truth image; and v represents the estimated image after denoising by the network. The solution for image denoising using the proposed loss function is represented as follows:

(10) $$\Theta^{*}=\underset{\Theta }{arg\min}\sum_{i}^{Num}Loss(u_{i},f(y_{i};\Theta ))$$

where f(·) denotes the mapping model that is used to estimate noise with its trainable parameters Θ under the loss function Loss(u,v).

## 4. Experiments on Public Datasets

### 4.1. Experimental Design

Currently, dedicated datasets for uncooled infrared image denoising are lacking. Therefore, the publicly available FLIR advanced driving assistance systems (ADAS) v2 dataset [36] was used. This dataset is a thermal infrared and visible light dual-light dataset for ADAS, with thermal infrared images captured by an FLIR Tau 2 long-wave uncooled infrared camera. The image size is 640 × 512 and contains 10,472 training images and 1144 validation images. The dataset includes various scenes, such as lanes, vehicles, street views, pedestrians, and plants, providing a rich variety of scenes suitable for denoising tasks. To create a denoised image dataset, images were selected from the ADAS v2 training set according to the criteria of fewer repeated scenes, lower noise levels, and weaker motion blur; 800 images were chosen for training and 60 images were chosen for testing from the test set.

The selected training and test datasets had relatively weak stripe noise; therefore, the BM3D denoising method was used to denoise the dataset and obtain ground-truth images without noise. Based on the proposed Gaussian and stripe noise mixed model, Gaussian and stripe noises were then added to the ground truth images to create a synthetic noise dataset. The network training utilized two NVIDIA RTX 3090 graphics cards with a batch size of 128 and a learning rate of 1 × 10^−4^ during training. Additionally, an Adam network parameter optimizer was used.

### 4.2. Experimental Results

We compared the proposed DER-UNet with several state-of-the-art denoising methods, including several powerful image restoration networks (DnCNN [13], DRUnet [16], FFDNet [37], SwinIR [17], and NAFNet [38]), as well as the latest composite noise infrared image denoising network SMUNet [12] and IDTransformer [39]. The comparison results based on the peak signal-to-noise ratio (PSNR) and structure similarity index measure (SSIM) are presented in Table 1.

**Figure 4:**
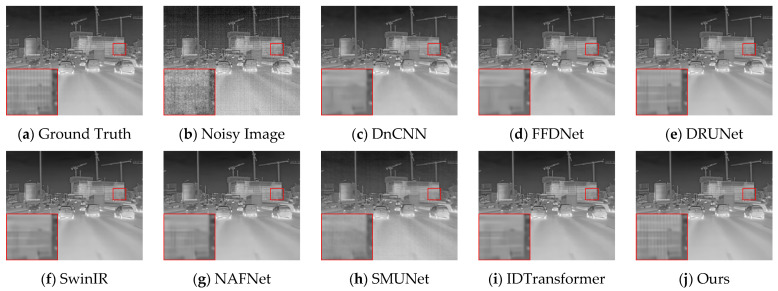
Denoising results for an architectural scene.

**Figure 5:**
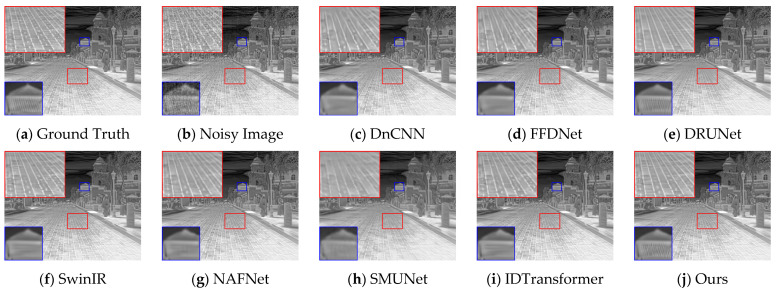
Denoising results for a street scene.

Figure 4 and Figure 5 present the denoising results of typical images from the test set, with the objective evaluation metrics, PSNR and SSIM, summarized in Table 2. The architectural details illustrated in Figure 4 suggest that the comparative methods tend to blur details in scenes rich in information. In contrast, DER-UNet effectively removes noise while preserving the target textures. In the street scene depicted in Figure 5, the DnCNN, SwinIR, and NAFNet networks manage to restore some road texture, while the DRUNet network recovers the roof texture. Notably, the proposed DER-UNet demonstrates superior preservation of both road and roof textures, closely resembling the ground truth image.

DER-UNet retains scene details and textures more effectively, achieving a visual effect closely resembling the ground truth image and performing best in PSNR and SSIM metrics. Among the comparison methods, the relatively strong DRUNet and SwinIR networks still lose some structural information from the original image. The PSNR and SSIM results support this finding, indicating alignment between visual assessments and objective metrics.

### 4.3. Ablation Study

An ablation study was conducted on the dual-encoder UNet to evaluate the contribution of the dual-encoder structure to the UNet network. Specifically, comparisons were made between the BSRA encoder single-branch UNet, the ESRA encoder single-branch UNet, and the complete DER-UNet network.

**Figure 6:**
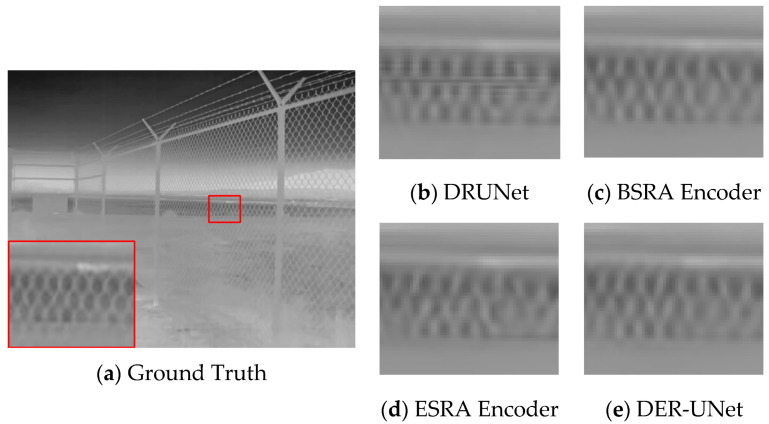
Comparative results of ablation study.

The results of the ablation experiments in Table 3 indicate that the dual-encoder structure of UNet shows improvements over the single-branch encoder structure across various metrics, with the DER-UNet achieving the best performance. A qualitative analysis of the ablation results, based on visual inspection of the dataset imagery (Figure 6), reveals further insights. The network employing the ESRA extended-scale encoder exhibits superior texture restoration capabilities; however, it also demonstrates a tendency to lose fine-scale details. Conversely, the basic DRUNet demonstrates insufficient texture recovery. The proposed DER-UNet, incorporating the dual-encoder structure, significantly enhances the recovery of detailed textures.

### 4.4. Network Runtime and Hardware Requirements

We compared DER-UNet with three representative networks, DRUNet, NAFNet, and SMUNet, in terms of runtime, floating-point operations per second (FLOPs), and memory requirements based on images sized 256 × 256 and 512 × 512. As indicated in Table 4, compared with image restoration networks DRUNet, NAFNet, and SMUNet, DER-UNet is not as advantageous in terms of runtime and FLOPs; however, it has better denoising results and is more suitable for denoising narrowband infrared images.

## 5. Experiments on Real Noise Dataset

### 5.1. Real Noise Dataset

Evidence from practice demonstrates that image denoising methods must be validated using real noisy images. Utilizing real noise datasets allows for the learning of the true noise characteristics of the camera, which has significant practical value. Currently, real noise datasets are primarily created by capturing and synthesizing actual multi-frame images to eliminate the effects of random noise, resulting in noise-free images that closely resemble reality. However, due to the necessity of employing a multi-frame collection method to obtain ground truth images, there is currently no publicly available denoising dataset specifically for uncooled infrared images.

**Figure 7:**
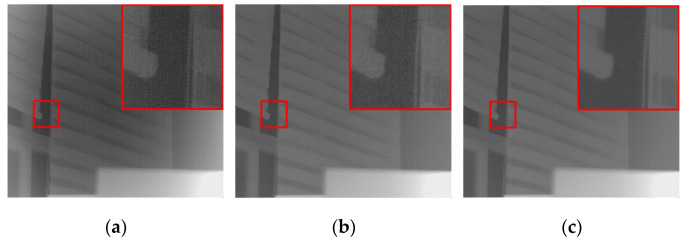
Process of uncooled infrared image noise: (**a**) uncooled infrared image noise; (**b**) image noise following internal baffle calibration; (**c**) image noise after averaging multiple frames.

In contrast to the methods for obtaining real noise in visible light images, uncooled infrared image noise comprises both Gaussian noise and stripe noise components (as shown in Figure 7a), making complete removal of all noise components through multi-frame acquisition impossible. In the mixed noise of uncooled infrared images, Gaussian noise and temporal stripe noise can be largely eliminated with multi-frame acquisition, while spatial stripe noise appears as fixed pattern noise that integrates into the background signal after collection. To obtain ground truth images from the real noise captured by uncooled infrared cameras, averaging multiple frames is typically employed to remove Gaussian noise and temporal stripe noise. However, most uncooled infrared camera cores are equipped with in-camera calibration that eliminates much of the spatial stripe noise before image acquisition (as illustrated in Figure 7b). As a result, the real noisy images produced are largely free of Gaussian noise and temporal stripe noise, retaining only a small amount of residual spatial stripe noise (as shown in Figure 7c), which can be utilized as a real noise dataset.

Images were captured using the iRaytek LA6110 uncooled infrared camera (IRay Technology Co., Ltd., Yantai, China), which features a 40 mm focal length lens and a narrowband filter with a wavelength range of 7–8.75 μm. Under outdoor natural conditions, a total of 220 sets of real noise data sequences were collected. Near-ground truth images were generated through multi-frame stacking and averaging, resulting in the creation of a real noise dataset. Of these, 200 image pairs were designated for network training, while 20 image pairs were set aside for network testing. Additionally, a subset of noisy images containing gas plume targets was collected to evaluate the denoising performance of the network trained on the real noise dataset specifically for gas plume images.

### 5.2. Narrowband Infrared Image Denoising Results

**Figure 8:**
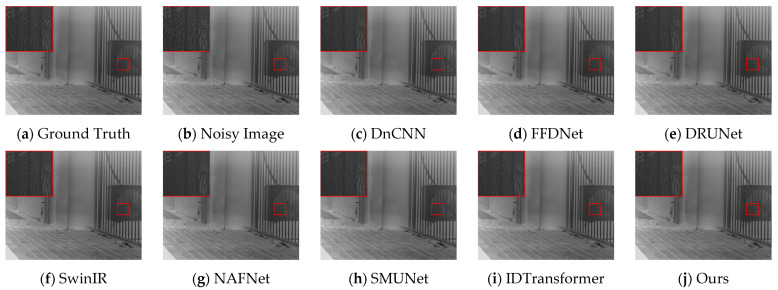
Denoising results for the real scene.

The proposed DER-UNet network was trained and tested, and comparisons were made with the DnCNN, FFDNet, DRUNet, SwinIR, NAFNet, SMUNet, and IDTransformer methods. The denoising results for the various methods are presented in Table 5. Figure 8 illustrates the denoising results for typical images from the test set. In scenes characterized by complex detail textures, DER-UNet demonstrates a superior ability to retain details compared to other methods, achieving better detail retention and higher performance metrics.

**Figure 9:**
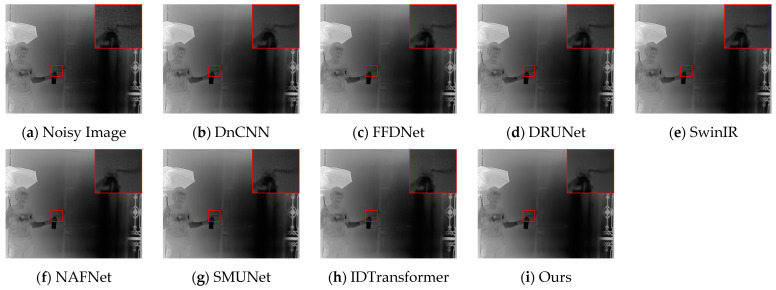
Denoising results for gas plume image.

Figure 9 illustrates the denoising results for images of gas plume. Because gas plume targets do not have a fixed shape, it is not feasible to obtain a ground truth image using multi-frame averaging methods. Therefore, this section provides only a subjective evaluation of the denoising results for various methods. DER-UNet shows superior performance in both noise removal and the retention of gas plume targets.

**Figure 10:**
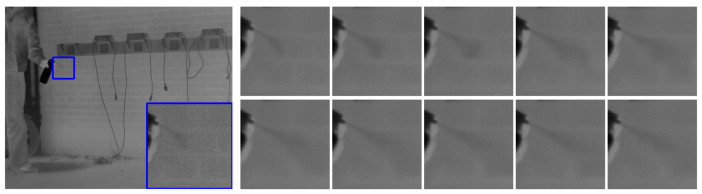
Denoising results of the gas plume image sequence.

Figure 10 presents the denoising results of the DER-UNet network applied to a sequence of actual gas plume images collected in the field, illustrating the network’s practical effectiveness. The left side of Figure 10 shows the original collected image alongside a magnified view of the noise in a local area of the plume. The right side displays a sequence of magnified local areas of the gas plume after denoising the subsequent 10 frames. The comparison of images before and after denoising, along with the results from multiple frames, highlights the reliability of the DER-UNet network in denoising sequential images.

## 6. Conclusions

To address the issue of image noise affecting uncooled infrared imaging detection of industrial hazardous gas leaks, we developed a dual-encoder denoising network based on UNet, inspired by the concept of dual-scale feature extraction for Gaussian and stripe noise. This network utilizes two branches: a basic residual attention CNN encoder for Gaussian noise and an expanded scale residual attention encoder for stripe features. This design effectively targets both types of noise components, enhancing the network’s performance and noise removal capability. We conducted training and testing on both a public dataset and a narrowband infrared image dataset. The results show significant performance advantages and improved image restoration effects compared to powerful image restoration networks such as DRUNet and SwinIR. Ablation experiments validate the effectiveness of the dual-encoder structure.

The dual-scale dual-encoder UNet architecture is well-suited for removing mixed noise from both local and non-local sources, effectively enhancing the signal-to-noise ratio of narrowband infrared images. After further optimization for speed, this approach is applicable for infrared imaging detection of industrial gas leak plumes and has broad potential in fields such as ultraviolet imaging and medical imaging.

## Figures and Tables

**Table 1:** Average PSNR and SSIM results for test set images. The best two values in each metric are denoted in red and blue, respectively.

Noise Level	Method	DnCNN	FFDNet	DRUNet	SwinIR	NAFNet	SMUNet	IDTransformer	Ours
15	PSNR	35.83	35.62	36.98	36.93	36.71	35.81	36.89	37.11
	SSIM	0.9426	0.9421	0.9544	0.9535	0.9525	0.9475	0.9536	0.9560

**Table 2:** PSNR and SSIM results for Figure 4 and Figure 5. The best two values in each metric are denoted in red and blue, respectively.

	Method	DnCNN	FFDNet	DRUNet	SwinIR	NAFNet	SMUNet	IDTransformer	Ours
Figure 4	PSNR	37.02	36.84	38.62	38.52	38.27	37.01	38.51	38.82
	SSIM	0.9581	0.9582	0.9707	0.9693	0.9682	0.9579	0.9696	0.9722
Figure 5	PSNR	31.72	31.56	32.64	32.94	32.53	31.74	32.74	33.04
	SSIM	0.8759	0.8747	0.9030	0.9081	0.8999	0.8789	0.9046	0.9114

**Table 3:** Ablation study and results.

Network	PSNR	SSIM
DRUNet	36.98	0.9544
BSRA Encoder UNet	37.04	0.9549
ESRA Encoder UNet	36.90	0.9537
DER-UNet	37.11	0.9560

**Table 4:** Execution time and hardware requirements. The best two values in each metric are denoted in red and blue, respectively.

Network	Image Size	DRUNet	NAFNet	SMUNet	Ours
Runtime (Seconds)	256 × 256	0.023	0.029	0.034	0.057
	512 × 512	0.074	0.064	0.118	0.175
Flops (Glops)	256 × 256	211.1	29.3	285.3	392.2
	512 × 512	844.3	117.4	1141.3	1568.6
Memory (GB)	256 × 256	2.69	4.19	3.99	4.45
	512 × 512	4.39	9.29	9.47	10.16

**Table 5:** Average PSNR and SSIM results for test set images. The best two values in each metric are denoted in red and blue, respectively.

Noise	Method	DnCNN	FFDNet	DRUNet	SwinIR	NAFNet	SMUNet	IDTransformer	Ours
Real Noise	PSNR	36.72	36.67	36.80	36.83	36.81	36.62	36.72	36.89
	SSIM	0.8969	0.8974	0.8994	0.8994	0.8993	0.8960	0.8971	0.9008

## Data Availability

Data underlying the results presented in this paper are not publicly available at this time but may be obtained from the authors upon reasonable request.
